# Tdp1 protects from topoisomerase 1–mediated chromosomal breaks in adult zebrafish but is dispensable during larval development

**DOI:** 10.1126/sciadv.abc4165

**Published:** 2021-01-29

**Authors:** Ringaile Zaksauskaite, Ruth C Thomas, Freek van Eeden, Sherif F. El-Khamisy

**Affiliations:** 1 Healthy Lifespan Institute, Sheffield Institute for Neuroscience, Department of Molecular Biology and Biotechnology, University of Sheffield, Sheffield S10 2TN, UK.; 2 Bateson Centre, Department of Biomedical Sciences, University of Sheffield, Sheffield S10 2TN, UK.; 3 The Institute of Cancer Therapeutics, University of Bradford, Bradford BD7 1DP, UK.

## Abstract

Deficiency in the DNA end-processing enzyme, tyrosyl-DNA phosphodiesterase 1 (TDP1), causes progressive neurodegeneration in humans. Here, we generated a *tdp1* knockout zebrafish and confirmed the lack of TDP1 activity. In adulthood, homozygotes exhibit hypersensitivity to topoisomerase 1 (Top1) poisons and a very mild locomotion defect. Unexpectedly, embryonic *tdp1*^−/−^ zebrafish were not hypersensitive to Top1 poisons and did not exhibit increased Top1-DNA breaks. This is in contrast to the hypersensitivity of Tdp1-deficient vertebrate models reported to date. Tdp1 is dispensable in the zebrafish embryo with transcript levels down-regulated in response to Top1-DNA damage. In contrast, *apex2* and *ercc4* (*xpf*) transcripts were up-regulated. These findings identify the *tdp1^−/−^* zebrafish embryo as the first vertebrate model that does not require Tdp1 to protect from Top1-DNA damage and identify *apex2* and *ercc4* (*xpf*) as putative players fulfilling this role. It highlights the requirement of distinct DNA repair factors across the life span of vertebrates.

## Teaser

The repair of protein-linked chromosomal breaks requires distinct factors throughout the life span.

## INTRODUCTION

Defects in DNA repair are linked to a variety of human disorders (*1*, *2*). DNA repair protects from cancer, neurological disorders, immunodeficiency, and premature aging. One example is spinocerebellar ataxia with axonal neuropathy 1 (SCAN1), which is caused by an autosomal recessive mutation in tyrosyl-DNA phosphodiesterase 1 (TDP1), primarily causing progressive cerebellar atrophy, neuropathy, and distal muscle weakness (*3*). This leads to the development of an ataxic gait, areflexia, dysarthria, loss of vibration sensation, and confinement to a wheelchair by early adulthood. TDP1 repairs a variety of damaged 3′ termini, namely 3′-phosphoglycolate, 3′-deoxyribose phosphate, 3′-histidine, and, importantly, 3′-topoisomerase 1 cleavage complexes (Top1-CCs) (*4*–*7*). Studies in yeast and chicken, as well as studies using recombinant human TDP1, have shown that TDP1 also plays a role in the repair of 5′-phosphotyrosyl lesions, caused by Top2-CCs (*8*, *9*). Top1-CCs occur because of the abortive activity of topoisomerase 1 (TOP1).

TOP1 relieves torsional stresses of DNA by transiently nicking one DNA strand and creating a phosphodiester bond between its active site tyrosine and the DNA, allowing controlled rotation of the nicked strand (*10*). The topoisomerase-DNA intermediate is called a TOP1-CC and can turn into a persistent single-strand break or double-strand break (DSB) because of collision with replication, transcription machinery, or the presence of proximal oxidative or bulky lesions (*11*–*15*). Out of all the lesions that TDP1 repairs, TOP1-CCs are the preferred substrates and have received the most interest because of their antireplicative properties, which can be used in cancer therapy by employing agents that stabilize TOP1-CCs, such as camptothecin (CPT) and its water-soluble analog topotecan (TPT) (*16*).

TDP1 cleaves the phosphodiester bond between the DNA and topoisomerase 1 by first generating a TDP1-DNA intermediate, which is then hydrolyzed using the histidine-493 active site (*6*, *17*). The H493R mutation in SCAN1 not only reduces TDP1 activity by 25-fold but also causes the accumulation of TDP1-DNA complexes; thus, it may have neomorphic properties (*18*, *19*). It is unclear whether the SCAN1 phenotype arises because of a reduction in TDP1 activity and, thus, elevated TOP1-CC levels, accumulation of TDP1-DNA complexes, or both.

*Tdp1^−/−^* mouse models were generated by three separate groups with some evidence of mild cerebellar degeneration (*19*–*21*). Notably, *Atm^−/−^* mice also do not adequately recapitulate the most notable clinical phenotype in ataxia telangiectasia, which is neurodegeneration, raising the question whether mouse is the ideal organism to model human neurological disorders (*22*). In an attempt to study the physiological function of TDP1 at the whole organismal level, we used CRISPR-Cas9 to generate a zebrafish *tdp1^−/−^* model. Zebrafish are vertebrates with high genetic similarity to humans that offer external development, high fecundity, and larval transparency and reach sexual maturity quickly, allowing large-scale studies that were not previously possible in mammalian models. Here, we describe the zebrafish *tdp1^−/−^* model and characterize its phenotype. We show that adult, but not embryonic, *tdp1^−/−^* zebrafish exhibit hypersensitivity to TOP1 poisons and a very mild behavioral defect. Our findings indicate that in the embryos, *tdp1* is down-regulated and, instead, *apex2* and *ercc4* (*xpf*) are up-regulated in response to Top1 inhibition. This model will be a valuable tool for the generation of a humanized SCAN1 zebrafish and will form the basis for genetic and chemical modifier screens to unravel new physiologically relevant TOP1-mediated repair mechanisms and adjuvants for TOP1-targeting chemotherapeutics.

## RESULTS

### Generation and validation of *tdp1^−/−^* zebrafish

**Fig. 1:**
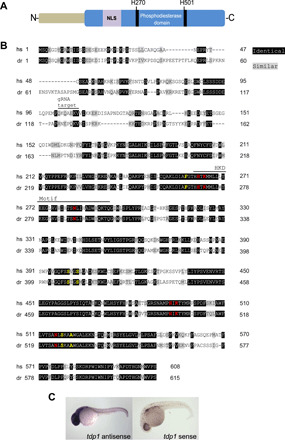
Zebrafish Tdp1 protein and its expression. (**A**) Schematic structure of zebrafish Tdp1 protein, depicting the nuclear localization signal (NLS), phosphodiesterase domain, and active site histidines H270 and H501 (*64*). Zebrafish H501 corresponds to human H493. (**B**) Alignment of *Homo sapiens* (hs) and *D. rerio* (dr) TDP1 protein sequence. Identical sequences are highlighted in black, and similar sequences are highlighted in gray. The sequence targeted for deletion and catalytic HKD motifs [HxK(x)_4_D(x)_6_GSxN] with catalytic sites (in red) are noted (*64*, *65*). Yellow denotes amino acids that contact the DNA substrate during repair. Accession numbers are as follows: hsTDP1, ENST00000335725.8, and drTdp1, ENSDART00000150149.2. Sequences were aligned using the Protein BLAST website: https://blast.ncbi.nlm.nih.gov/Blast.cgi?PAGE=Proteins. (**C**) Whole-mount in situ hybridization for *tdp1* mRNA in wild-type zebrafish at 24 hpf. Sense probes for *tdp1* mRNA were used as a negative control.

Similar to mammals, zebrafish contain a single *tdp1* ortholog. The zebrafish *tdp1* gene encodes a protein of 615 amino acids with a high degree of similarity to its human counterpart (Fig. 1, A and B). All five DNA-interacting amino acids and six catalytic residues are conserved. Whole-mount in situ hybridization revealed ubiquitous *tdp1* expression with an emphasis in the head in 24-hpf (hours post-fertilization) wild-type embryos (Fig. 1C).

**Fig. 2:**
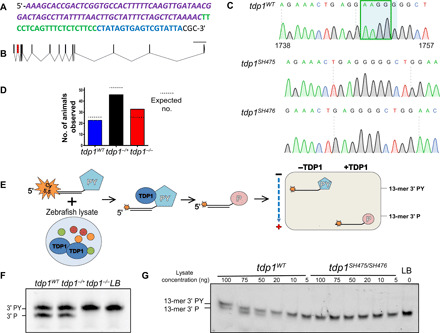
Generation and validation of *tdp1^−/−^* zebrafish using the CRISPR-Cas9 system. (**A**) Sequence of oligonucleotide used for guide RNA (gRNA) synthesis. The scaffolding sequence is in purple; the target sequence is in green, and the T7 polymerase promoter is in blue. (**B**) Intron-exon structure of the *D. rerio tdp1* gene. Exon 2 (in red) was targeted for mutation by Cas9; scale bar, 5000 bp. Photo credit: Ringaile Zaksauskaite, University of Sheffield (generated using http://wormweb.org/exonintron). (**C**) Sequences of the target region in *tdp1^WT^* zebrafish and two isolated deletion alleles, *tdp1^SH475^* and *tdp1^SH476^*. The 5-bp deletion (SH476; light blue box) and the 4-bp deletion (SH475; green square). (**D**) *Tdp1^−/+^* zebrafish were crossed and genotyped at adulthood; χ^2^ = 2.941 with two degrees of freedom; two-tailed *P* value of 0.5316. (**E**) Diagram depicting the TDP1 activity assay showing a 5′ labeled oligomer with a 3′-phosphotyrosyl (PY) that is incubated with zebrafish protein lysate. Active TDP1 processes the 3′-PY into a phosphate group, resulting in a band shift on a DNA sequencing gel. (**F**) TDP1 activity assay was performed on 600 ng of lysate from 4-dpf embryos. (**G**) TDP1 activity assay was performed on fin clips from adult zebrafish. LB, lysis buffer control

Two deletion alleles, SH475 and SH476, were generated in exon 2 of the zebrafish *tdp1* gene using the CRISPR-Cas9 system (Fig. 2, A to C). SH475 and SH476 contained a 4– and 5–base pair (bp) deletion, respectively, which both cause a frameshift and result in a putative early stop codon 21 and 6 amino acids downstream of the deletion, respectively (Fig. 2C). The founders carrying these alleles were out-crossed to a wild-type strain to create *tdp1^−/+^* zebrafish (*tdp1^SH475/+^* and *tdp1^SH476/+^*), which were then crossed to produce *tdp1^SH475/SH476^* and *tdp1^SH475/SH475^* (hereafter referred to as *tdp1^−/−^*) fish. *Tdp1^−/−^* animals were born at expected Mendelian ratios, had normal longevity, and appeared to be in good health (Fig. 2D). To confirm the loss of Tdp1 protein in the mutants, we used a TDP1 biochemical activity assay. In this assay, a labeled oligonucleotide harboring a 3′ phosphotyrosyl moiety (3′-PY) is incubated with whole embryo lysate. If active TDP1 is present, then a band shift on a DNA sequencing gel is observed (Fig. 2E). The band shift denotes the cleavage of the phosphodiester bond between the phosphate and tyrosine, which mimics the bond linking the tyrosine residue in TOP1 and the 3′ terminus of DNA. We incubated lysates from 4-dpf (days post-fertilization) embryonic and adult fish with such an oligonucleotide and did not observe a band shift in *tdp1^−/−^* samples, confirming that Tdp1 activity has been abolished by the mutation at 4 dpf (Fig. 2, F and G).

### Adult, but not embryonic, *tdp1^−/−^* zebrafish exhibit a very mild locomotion defect

**Fig. 3:**
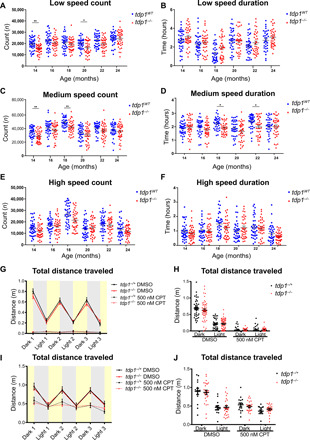
*Tdp1^−/−^* fish have a mild locomotion defect in adulthood but not at 4 to 5 dpf. (**A** to **F**) Adult zebrafish movement was recorded with a camera system for 6 hours. Time spent swimming at low (<30 mm/s) (A), medium (30 to 60 mm/s) (C), and high (E) speeds (60 mm/s or above) and count of times low (B), medium (D), and high (F) swimming speeds were plotted. Each of the 18 fish from either genotype was recorded twice (*n* = 36) and shown as average ± SEM. *P* values were calculated by two-tailed Student’s *t* test with Holm adjustment for multiple comparisons. (**G** to **J**) Four-dpf (G and H) and 5-dpf (I and J) embryos from a single female *tdp1^−/−^* and male *tdp1^−/+^* incross were subjected to 3 cycles of 5-min darkness and 5-min light using the photomotor response assay. (G and I) Total distance traveled in each cycle was plotted in each data point as average ± SEM. *P* value, two-tailed Student’s *t* test. (H and J) Total distance traveled in all light or dark cycles was measured. Lines indicate average ± SEM. (I) Total distance traveled each cycle was plotted as average ± SEM. *P* values were calculated using two-tailed Student’s *t* test. DMSO, dimethyl sulfoxide. *P < 0.05; **P < 0.01.

To assess potential progressive neurodegeneration in *tdp1^−/−^* zebrafish, behavioural analysis using a camera system was performed in *tdp1^−/−^* fish and *tdp1*^WT^ siblings every 2 months from 14 to 24 months of age (Fig. 3 and fig. S1). Parameters measured were the number of times each of the three speeds (low, medium, and high) were initiated (speed count), the length of time spent swimming at each speed (speed duration), total distance, and average speed. We defined low speed as <30 mm/s, medium speed as 30 to 60 mm/s, and high speed as 60 mm/s or above. Data analysis revealed a modest but significant reduction in two of six time points for low speed count, medium speed duration, and count in the *tdp1^−/−^* fish in comparison to wild types (Fig. 3). Although an overall trend of reduced locomotion was observed across all parameters, the difference between *tdp1^−/−^* fish and their wild-type siblings in low speed count, medium speed, high speed duration and count, total distance traveled, and average speed did not reach statistical significance (Fig. 3 and fig. S1, A and B). These data suggest a trend of mildly impaired locomotion across all parameters tested.

The fish were also subjected to swim tunnel analysis at 19 months of age (fig. S1, C to F). In this assay, the fish were placed in a tube filled with water and an increasing current was applied against them. Endurance of the fish can be determined by calculating the critical swimming speed, *U*_crit_, as described in Materials and Methods. Critical swimming speed is dependent on the percentage of fish still swimming at each flow rate, which was not affected in *tdp1^−/−^* fish (fig. S1D). Since the weight and length of the fish could affect the result, these parameters were measured among the fish tested and were found to be equal (fig. S1, E and F). Recovery after vestibular disorientation was also examined using the “drop test” (fig. S1, G to I). Fish were dropped into a deep tank from 10 cm above the water, and time spent at the bottom part of the tank, freezing duration, and number of transitions between the top and the bottom of the tank were recorded. Wild-type fish tend to first freeze at the bottom, then swim at the bottom of the tank, and later explore the top region (*23*). Any deviation from this typical response could indicate vestibular defects. However, no significant differences between wild-type and *tdp1^−/−^* fish were found in the duration of time spent in the bottom area of the tank (fig. S1G), number of transitions between top and bottom of the tank (fig. S1H), or freezing duration (fig. S1I).

To assess whether any ataxic phenotypes were present during development, we monitored the photomotor response of 4- and 5-dpf embryos with and without exposure to the TOP1 inhibitor, CPT. On the basis of the known function of *tdp1* and observations in mice, CPT treatment was expected to exacerbate otherwise mild phenotypes (*19*, *21*). *Tdp1^−/−^* females were crossed with *tdp1^−/+^* males to avoid any maternal contribution of *tdp1* mRNA in the progeny. The embryos were then treated with 500 nM CPT overnight and subjected to 5-min light/5-min dark cycles (Fig. 3, G to J), which have been shown to produce a highly robust pattern of larval movement (*24*–*28*). Measurement of total distance traveled by the larvae at each 5-min cycle (Fig. 3, G and I) and in all light and dark cycles combined (Fig. 3, H and J) revealed no significant differences between *tdp1^−/−^* and *tdp1^−/+^* siblings at 4 and 5 dpf.

### Adult *tdp1^−/−^* zebrafish are hypersensitive to Top1 poisons

**Fig. 4:**
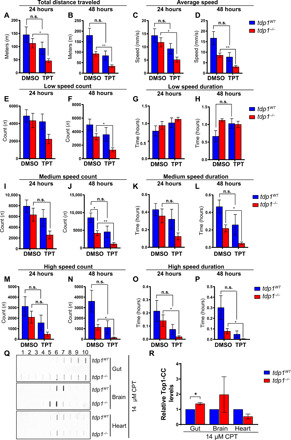
Adult *tdp1^−/−^* zebrafish are hypersensitive to TPT. (**A** to **P**) Zebrafish (27-month-old) were intraperitoneally injected with TPT (22.5 mg/kg) or DMSO (22.5 mg/kg) on two consecutive days for a final concentration of 45 mg/kg and monitored for 1.5 hours using a camera system 24 and 48 hours after the second injection. *P* values, two-tailed Student’s *t* test with Holm post hoc analysis for multiple comparisons. Total distance traveled (A and B), average speed (C and D), low speed count (E and F), low speed duration (G and H), medium speed count (I and J), medium speed duration (K and L), high speed count (M and N), and high speed duration (O and P) were quantified after the second injection. (**Q**) Tissues were dissected from 29-month-old fish and treated with 14 μM CPT for 2 hours and then examined for Top1-CC accumulation using CsCl fractionation and immunoblotting, as described in Materials and Methods. (**R**) Quantification of (Q); two biologically independent experiments for heart and gut and four for brain; for each independent repeat, either four hearts or four guts were pooled. One brain was used for each independent repeat; ±SEM. *P* values, two-tailed Student’s *t* test. n.s., not significant. *P < 0.05; **P < 0.01.

TDP1-deficient human, avian, and murine cells, as well as *Tdp1^−/−^* mice, are hypersensitive to topoisomerase-linked breaks induced by CPT and its clinical derivatives, TPT and irinotecan (*8*, *19*, *21*, *29*). To ascertain the role of Tdp1 in zebrafish following exposure to topoisomerase 1 poisons, 27-month-old animals were subjected to intraperitoneal injections of TPT. The fish received one daily injection of 22.5 mg/kg for two consecutive days, and their locomotion was monitored after each injection and then 24, 48, and 72 hours after the second injection (Fig. 4 and fig. S2). Locomotion analysis revealed a significant reduction in total distance traveled over 6 hours in TPT-treated *tdp1^−/−^* fish in comparison to dimethyl sulfoxide (DMSO)–injected *tdp1^−/−^* fish at 24 (Fig. 4A) and 48 hours (Fig. 4B) after the second injection. Likewise, a corresponding decrease of average speed was found in TPT-treated *tdp1^−/−^* fish in comparison to DMSO-injected *tdp1^−/−^* fish at 24 (Fig. 4C) and 48 hours (Fig. 4D) after the second injection. TPT treatment did not significantly affect low speed count at 24 hours (Fig. 4E) but markedly reduced it at 48 hours in TPT-treated *tdp1^−/−^* fish, in relation to control *tdp1^−/−^* fish (Fig. 4F). On the other hand, low speed duration was not altered because of the treatment (Fig. 4, G and H). Medium speed count was not affected at 24 hours (Fig. 4I) but strongly reduced at 48 hours in the TPT-treated *tdp1^−/−^* fish in comparison to DMSO-injected *tdp1^−/−^* fish (Fig. 4J). Likewise, differences in medium speed duration between TPT-treated and DMSO-treated *tdp1^−/−^* fish were not significant until 48 hours after the second injection (Fig. 4, K and L). Furthermore, no obvious differences were observed in high speed count at 24 hours after treatment (Fig. 4M), while at 48 hours, high speed count was reduced in TPT-treated *tdp1^−/−^* fish, in relation to control *tdp1^−/−^* fish (Fig. 4N). High speed duration was significantly lower at both the 24- and 48-hour time points in the TPT-treated *tdp1^−/−^* fish in comparison to DMSO-injected *tdp1^−/−^* fish (Fig. 4, O and P). All locomotion parameters were also quantified 72 hours after the second injection, but no significant differences were found (fig. S2). To summarize, TPT treatment significantly decreased low speed count, medium speed duration, and high speed duration and count in *tdp1^−/−^* animals at 48 hours after the second injection. High speed duration was also lower in TPT-treated *tdp1^−/−^* fish than in DMSO-treated *tdp1^−/−^* fish at 24 hours after the second injection. Notably, none of the locomotion parameters were significantly different in wild-type siblings before and after treatment at this dose, showing that adult *tdp1^−/−^* zebrafish are hypersensitive to increased Top1-CCs.

To confirm TPT hypersensitivity in adult *tdp1^−/−^* zebrafish, we dissected the gut, brain, and heart from 29-month-old fish and treated the tissues with CPT (Fig. 4, Q and R). Tissue lysates were subjected to cesium chloride fractionation to purify Top1-linked DNA breaks. CPT treatment caused a significant increase in Top1-CCs in the gut of *tdp1^−/−^* zebrafish in comparison to their wild-type siblings, but there were no significant differences in the brain or heart tissues. Increased Top1-CC level in the gut of CPT-treated adult *tdp1^−/−^* zebrafish is consistent with the observed reduced mobility phenotype after TPT treatment.

### Embryonic *tdp1^−/−^* zebrafish are not hypersensitive to Top1 poisons

**Fig. 5:**
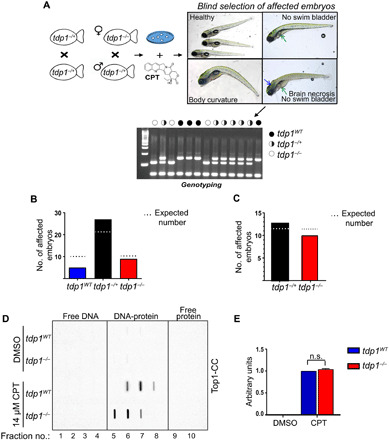
*Tdp1^−/−^* zebrafish embryos are not hypersensitive to CPT. (**A**) Diagram of blind assay to assess CPT sensitivity showing embryos with severe body curvature or brain necrosis or lacking swim bladders. (**B**) *Tdp1^−/+^* fish were incrossed, and at 4 dpf, sibling embryos were treated with CPT (350, 500, and 750 nM) overnight. At 5 dpf, the most strongly affected embryos were blindly selected and genotyped. χ^2^ = 4.902 with two degrees of freedom. The two-tailed *P* value is equal to 0.0862. (**C**) A female *tdp1^−/−^* fish was crossed with a male *tdp1^−/+^* fish, and at 4 dpf, the embryos were treated with 500 and 1 μM CPT overnight. At 5 dpf, most strongly affected siblings were blindly selected at each concentration and genotyped. χ^2^ = 0.391 with one degree of freedom. The two-tailed *P* value is equal to 0.5316. (**D**) Three-dpf embryos were treated with 14 μM CPT for 2 hours; then, TOP1-CC was examined using CsCl fractionation and immunoblotting, as described in Materials and Methods. (**E**) Quantification of (D); three biologically independent experiments, ±SEM. *P* values, two-tailed Student’s *t* test.

We next wondered whether embryonic *tdp1^−/−^* zebrafish would also be hypersensitive to Top1 poisons. *Tdp1^−/+^* fish were incrossed, and the progeny was treated with various concentrations of CPT at 4 dpf for 16 hours. As each concentration produced a range of phenotypes at 5 dpf, the fish exhibiting the most severe phenotypes, such as body curvature, brain necrosis, and lack of swim bladder were blindly selected and genotyped (Fig. 5A). From previous studies, we predicted that the most severely affected embryos should be positive for *tdp1* mutation (*19*, *21*, *30*). In notable contrast to our prediction, genotyping revealed a Mendelian distribution of genotypes across the selected embryos (Fig. 5B). The lack of hypersensitivity of *tdp1^−/−^* embryos could be a result of maternal contribution of *tdp1* mRNA. To address this possibility, we repeated this experiment by crossing female *tdp1^−/−^* fish to male *tdp1^−/+^* fish. Unexpectedly, the genotypes blindly selected this way were also consistent with Mendelian ratios from such a cross (Fig. 5C). To ascertain whether CPT was indeed inhibiting topoisomerase 1 in zebrafish, Top1-CC induction in 3-dpf *tdp1^−/−^* embryos was measured by cesium chloride fractionation of lysates into free protein, DNA-protein complexes and free DNA after a 2-hour CPT treatment (Fig. 5, D and E). Immunoblotting of fractions with a Top1-CC antibody revealed that CPT did induce Top1-CCs in equal levels in *tdp1^−/−^* and *tdp1^WT^* fish. Together, these results suggest that zebrafish embryos, but not adults, can tolerate Top1-induced DNA breaks despite Tdp1 loss.

### Embryonic *tdp1^−/−^* fish do not exhibit increased CPT-induced DNA double-stranded breaks

Top1-CCs could be converted into DNA DSBs during replication. Therefore, we measured overall and local phosphorylated H2A histone family member X (γH2AX) levels in *tdp1^−/−^* embryos after CPT and ionizing radiation (IR) treatment. IR primarily causes DNA strand breaks and base modifications, both of which can trap Top1 on DNA (*31*, *32*). First, 4-dpf embryos were treated with CPT overnight and then harvested for immunoblotting for γH2AX at 5 dpf (fig. S3, A and B). An induction of γH2AX was observed in both genotypes, without a significant increase in *tdp1^−/−^* embryos. The embryos were also treated with irradiation at 24 hpf, and overall γH2AX levels were analyzed by immunoblotting; however, similar levels of γH2AX were observed (fig. S3, C and D). As the cerebellum is selectively vulnerable in SCAN1 patients and mice, local induction of γH2AX foci in the 24-hpf cerebellum and optic tectum was also quantified. Embryos (24 hpf) were irradiated with 22 gray and fixed either immediately or after a 30-min recovery for immunofluorescence analysis (fig. S3E). Quantification of γH2AX foci revealed no significant differences in IR-induced DSBs between genotypes in both tissues (fig. S3F).

### Tdp1 is dispensable for Top1-CC repair in the zebrafish embryo

**Fig. 6:**
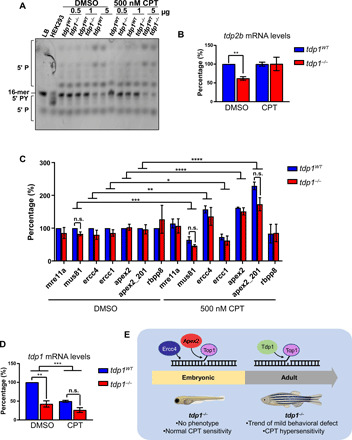
Tdp1 is not required for Top1-CC repair in zebrafish embryos. (**A**) Four-dpf zebrafish were treated with 500 nM CPT overnight, and lysates were incubated with a 3′ labeled oligonucleotide with a 5′-phosphotyrosyl (PY). TDP2 processes the phosphotyrosyl moiety into a phosphate group, resulting in a lower band on a DNA sequencing gel. We noted bands that are higher than the original substrate, which suggest further repair events taking place using the zebrafish lysate. Lysis buffer (LB) was used as a negative control and human embryonic kidney (HEK) 293 cell lysate as a positive control. (**B** to **D**) Four-dpf zebrafish embryos were treated with 500 nM CPT overnight, and total RNA was processed by reverse transcription qPCR (RT-qPCR). Transcript levels normalized to *rps29* are shown; three biologically independent experiments, ±SEM. *P* values, two-way analysis of variance (ANOVA) with Holm post hoc analysis for multiple comparisons. (**E**) A model for the requirement of distinct DNA repair factors for Top1-CC repair during the zebrafish life span. We propose that during embryonic development, zebrafish use Apex2 and Ercc4 to repair Top1-induced DNA breaks. During adulthood, however, Tdp1 becomes essential for repairing Top1-CCs, and thus, *tdp1^−/−^* fish develop a mild behavioral defect and CPT hypersensitivity in adulthood but not at embryonic stage. *P < 0.05; **P < 0.01; ***P < 0.001; and ****P < 0.0001.

The hypersensitivity to Top1 poisons in adult zebrafish, but not embryos, suggests the presence of a compensatory pathway during the embryonic stages, which could be either overwhelmed or inaccessible during adulthood. It has been shown that Tdp2 can repair Top1-CCs in the absence of Tdp1 (*33*). To investigate the possibility of compensation by Tdp2, we treated 4-dpf embryos with 500 nM CPT overnight and harvested the embryos for protein and RNA analyses. We chose a low dose of CPT (500 nM) for a longer time (16 hours) over a shorter pulse with a high dose to observe the long-term effects on transcription. Embryo lysates were incubated with a labeled oligonucleotide with a 5′ phosphotyrosyl moiety, mimicking Top2-CCs, and then run on a DNA sequencing gel, where a band shift indicates Tdp2 activity. No overt differences were observed between wild-type and *tdp1^−/−^* embryos (Fig. 6A). The RNA was transcribed into cDNA and used for quantitative polymerase chain reaction (qPCR) analysis of mRNA levels. Contrary to expectations, *tdp2b* mRNA was significantly reduced in DMSO-treated *tdp1^−/−^* embryos (Fig. 6B). This difference, however, seemed to be abrogated by CPT whereby *tdp2b* mRNA exhibited similar levels in *tdp1^−/−^* and *tdp1^WT^* embryos. Thus, *tdp2b* is not the responsible compensatory factor. We then tested the next most likely compensation candidates *mre11a*, *mus81*, *ercc1-ercc4*, *apex2*, and *rbbp8*. These endonucleases have been shown to have the ability for Top1-CC repair in mouse, chicken, and yeast cells (*34*–*39*). We treated 4-dpf embryos with 500 nM CPT overnight for 16 hours and harvested RNA at 5 dpf. The RNA was transcribed into cDNA, which was used as a template in qPCR (Fig. 6C). Two different primer pairs were used for *apex2*: one targeting both transcript 201 (*ENSDART00000021514.7*) and 202 (*ENSDART00000189272.1*), denoted *apex2*, and another one targeting only one transcript 201, denoted *apex2_201*. qPCR results showed no significant difference in the expression of *mre11a*, *mus81*, *ercc1*, *ercc4*, *apex2*, and *rbbp8* between *tdp1^−/−^* and *tdp1^WT^* embryos both in DMSO- and CPT-treated conditions. However, we did note a remarkable increase in *apex2* and *ercc4* mRNA after CPT treatment in both genotypes. There was a significant increase in expression of *apex2* using both primer pairs; however, transcript 201 showed an even higher increase than transcripts 201 and 202 together. We also observed that CPT treatment resulted in a significant decrease in *ercc1* and *mus81*.

To examine the mechanism/s that enable zebrafish embryos to tolerate Tdp1 loss, we conducted microarray analyses comparing gene expression profiles of CPT-treated *tdp1^−/−^* and *tdp1^WT^* embryos (fig. S4). RNA was extracted from 5-dpf embryos after an overnight 500 nM CPT treatment and processed for microarray investigations. Data analysis revealed 1720 transcript clusters that were differentially expressed between *tdp1^−/−^* and *tdp1^WT^* embryos (fig. S4A, B). A total of 1021 of these transcript clusters were up-regulated, and 699 were down-regulated. As expected, *tdp1* was significantly down-regulated in *tdp1^−/−^* fish (fig. S4C). The top three hits with the lowest *P* value were zinc finger protein 644 (*znf644b*), *si:dkeyp-53e4.4* (Ensembl accession: ENSDARG00000114935), and *CABZ01069162.1* (Ensembl accession: ENSDARG00000115161). *Znf644b* and *si:dkeyp-53e4.4* were up-regulated by 4.88- and 10.13-fold, respectively, while *CABZ01069162.1* was down-regulated by 6.54-fold. *Znf644b* is one of two genes encoding for the Znf644 transcription factor in zebrafish (*40*). It contains five C2H2 and one atypical zinc finger motif. Znf644 regulates H3K9-mediated gene silencing during neurogenesis and has been linked to autosomal dominant high-grade myopia (*40*–*42*). *Si:dkeyp-53e4.4* and *CABZ01069162.1* are uncharacterized fish-specific genes. *CABZ01069162.1* belongs to the TF613015 PiggyBac transposable element-derived family. The expression of *sprtn* and *neil1* was significantly increased in *tdp1^−/−^* embryos (fig. S4C). Sprtn is a DNA-dependent metalloprotease that plays a role in the resolution of DNA-protein cross-links, including Top1-CCs, while Neil1 is a glycosylase that participates in the first step of base excision repair (*43*–*46*). As *sprtn* and *neil1* were the most likely compensation candidates from the microarray screen, we carried out qPCR to confirm these hits, as described previously for *tdp2b* (fig. S4D). qPCR results showed a decrease in *sprtn* and *neil1* in *tdp1^−/−^* embryos in comparison to wild types, both after DMSO and CPT treatment. This decrease was only significant for *neil1*. qPCR also showed a significant decrease in *neil1* expression after CPT treatment.

The fact that none of the key Top1-CC repair enzymes were up-regulated in the *tdp1^−/−^* embryos led us to consider the possibility that Tdp1 is not required for Top1-CC repair at this stage. We thus investigated the expression of *tdp1* mRNA after incubation with CPT. We treated 4-dpf embryos with 500 nM CPT overnight for 16 hours and harvested RNA at 5 dpf. As expected, *tdp1* mRNA was reduced by more than 50% in DMSO-treated *tdp1^−/−^* embryos in comparison to wild-type siblings (Fig. 6D). To our surprise, however, *tdp1* expression was significantly reduced after incubation with CPT, suggesting that *tdp1* may not be required for Top1-CC repair in zebrafish embryos. In contrast, the expression of *ercc4* and *apex2* was increased following CPT treatment (Fig. 6C). We next generated *apex2* crispants whereby mosaic mutations are introduced in the target gene using CRISPR-Cas9 to obtain a stable knockdown of the protein. A pool of four guide RNAs (gRNAs) designed across the *apex2* gene was injected before or at the one-cell stage. PCR assessment of gRNA efficiency revealed an average 42% of detectable mutations (fig. S5, A and B). Treatment of the 4-dpf *apex2* crispant embryos with CPT revealed no overt morphological differences when compared to controls (fig. S5, C and D). In contrast, recording embryo movement with a camera followed by quantification of the total distance traveled revealed a putative role for *apex2* in response to Top1-CC. Whereas *apex2* gRNA injections did not affect the movement of DMSO-treated embryos, it significantly reduced the total distance traveled in CPT-treated *apex2* crispants in comparison to CPT-treated controls (fig. S6E). These data suggest a role for *apex2* in repairing Top1-CCs in zebrafish embryos, which is consistent with recently reported in vitro biochemical assays and studies in human cells (*38*, *39*), and lay the foundation of further validations in follow-up studies.

## DISCUSSION

SCAN1 is caused by an active site mutation in *TDP1* that leads to progressive neurodegeneration. It is thought that neurodegeneration is a combined result of the accumulation of protein-linked DNA breaks, a lack of alternative repair pathways, high oxidative stress, and transcription levels in postmitotic neurons (*47*). However, studies show that TDP1 is also required in replicating cells under genotoxic stress (*29*, *48*, *49*). Mouse *Tdp1*^−/−^ models have been generated in attempts to unravel the physiological role of TDP1, but only a few mild phenotypes were reported in adult animals, such as a slight reduction in brain-to-body ratio and hypoalbuminemia (*19*–*21*). No overt ataxia was observed. Two groups also generated *Drosophila melanogaster* knockouts of *glaikit*, a *Tdp1* ortholog; however, their results were conflicting. Dunlop *et al*. (*50*) demonstrated that deletion of *glaikit* (*gkt*) in flies leads to defective neurogenesis and, thus, embryonic lethality, whereas Guo *et al*. (*30*) found that the flies are viable with little phenotype other than a decreased life span in females. As the first group linked their findings to a disruption of membrane trafficking and did not directly assess *tdp1* activity, it is thought that the phenotype is unrelated to *tdp1*.

Here, we describe the first *tdp1* knockout zebrafish. We show modest reductions in the number of times adult *tdp1^−/−^* fish initiated low and medium speeds and the duration of time they spent swimming at medium speed, suggesting a mild behavioral deficiency. However, such reductions were only statistically significant in a few of the recorded time points, which is likely due to subtle differences. Nevertheless, there was a trend of reduced movement throughout the analyses. Previous studies also found hypersensitivity to Top1 poisons or the non-CPT inhibitor NSC-725776 in adult mice and flies, which our findings in TPT-treated adult fish corroborate (*19*, *21*, *30*). We found that most locomotion parameters were significantly affected in adult *tdp1^−/−^* fish either 24 or 48 hours after administering TPT (45 mg/kg). This was concomitant with increased levels of Top1-CCs in the gut of adult *tdp1^−/−^* fish in comparison with their wild-type siblings after TPT treatment. Although we predicted postmitotic tissues, such as the heart and the brain, to be predominantly vulnerable to loss of Tdp1, we did not observe significant differences in Top1-CC levels in these tissues between adult *tdp1^−/−^* and *tdp1^WT^* fish after CPT treatment. This could be due to a preferential effect of acute exposure to CPT in replicating tissues compared to noncycling tissues (*21*, *51*, *52*). We did not observe an ataxic phenotype in *tdp1^−/−^* larvae, which was not unexpected given that patients with SCAN1 do not start showing symptoms until late childhood (*3*). Next, we assessed the sensitivity to CPT in embryonic *tdp1^−/−^* zebrafish and, notably, found that, in contrast to studies in mice, human, and avian cells, *tdp1^−/−^* embryos were not hypersensitized to CPT. We note that our results here are reminiscent of a recent report in plants showing that *Arabidopsis Tdp1* knockout is also not hypersensitive to TOP1-induced DNA damage (*53*). At the molecular level, there were no differences in Top1-CC and γH2AX levels between *tdp1^−/−^* and wild-type embryos either before or after CPT or IR treatment, confirming that loss of Tdp1 had no measurable effect in embryos, even under challenging conditions. We have found the lack of CPT sensitivity to be corroborated by an unexpected down-regulation of *tdp1* mRNA after CPT treatment, suggesting that *tdp1* is not required to repair Top1-CCs in zebrafish embryos. Instead, we show that *apex2* and *ercc4* are up-regulated. While *apex2* crispants did not show overt morphological differences following CPT treatment, they exhibited a significant reduction in total distance traveled. This adds weight to the notion that *apex2* is responsible for Top1-CC repair in zebrafish embryos. Although these findings need further experimental proof in follow-up studies, they are consistent with recent reports showing a role for *apex2* in TOP1-CC repair. For a while, it has been known that the yeast *apex2* ortholog *apn2* has phosphodiesterase activity (*53*–*55*). Only recently, it was shown that the vertebrate Ape2 has the unique ability to process phosphotyrosine-DNA conjugates into readily ligatable DNA ends, which our data corroborate (*38*, *39*). *Ercc4* would also be an intriguing candidate for Top1-CC repair; however, we found *ercc1*, an essential part of the *ercc1-ercc4* heterodimer, to be slightly down-regulated after CPT treatment. It is likely that zebrafish Ercc4 does not require Ercc1 for DNA binding and activity, which could explain this conundrum. It is reminiscent of Ercc4 in archaea, which do not have Ercc1 at all (*56*). *Mus81* and *neil1* were significantly down-regulated as well. This result indicates that, as well as *tdp1*, these factors are likely not required for Top1-CC repair in the zebrafish embryo. *Tdp2b*, *mre11a*, and *rbbp8* (*ctip*) did not show significant expression changes in response to increased Top1-CCs, and *tdp2* did not show a measurable increase in activity. Despite the lack of a phenotype or CPT hypersensitivity in *tdp1^−/−^* embryos, microarray analysis identified gross transcriptional changes in CPT-treated *tdp1^−/−^* embryos in comparison to wild types. This included a slight ~1.3-fold increased expression of *sprtn* and *neil1* in *tdp1^−/−^* embryos. However, qPCR validation showed a down-regulation in *neil1*, both before and after CPT treatment, and a nonsignificant trend of *sprtn* down-regulation in *tdp1^−/−^* embryos.

To summarize, we have generated and extensively characterized a *tdp1^−/−^* zebrafish mutant, which is hypersensitive to Top1 poisons and has a very mild locomotion defect in adulthood. We found that Tdp1 in zebrafish embryos does not appear to play a role in Top1-CC repair, which is corroborated by the lack of hypersensitivity to Top1 poisons at this stage. We show that *apex2* and *ercc4* are up-regulated in response of CPT treatment and are, thus, the factors that are most likely repairing this type of damage in the zebrafish embryo. Our findings are exciting because the lack of Tdp1 requirement to cope with Top1 poisons has only been observed in *Arabidopsis thaliana* (*57*), thereby identifying the zebrafish embryo as the first vertebrate model that does not require Tdp1 to protect from TOP1-mediated DNA damage. We propose *apex2* and *ercc4* (*xpf*) as primary players protecting from TOP1-induced damage in zebrafish embryos and suggest the utility of their inhibition as adjuvants to Top1-targeting chemotherapeutics.

## MATERIALS AND METHODS

### Zebrafish husbandry

Zebrafish (*Danio rerio*) were housed in the Bateson Centre aquaria and fed *Artemia* or dry food (Gemma Micro, SKRETTING). Zebrafish were kept at a constant temperature of 28°C and 14-hour on/10-hour off light cycle. All work were performed in accordance with the U.K. Home Office Animals (Scientific Procedures) Act 1986 under personal license I023015BA held by R.Z. and project licenses PB2866EDO and PC39B259E held by F.v.E.

### Whole-mount in situ hybridization

Whole-mount in situ hybridization was carried out as previously described (*58*). A probe of 1030 bp was amplified with KOD Hot Start DNA polymerase (Merck, 71086) from zebrafish cDNA using primers listed in Table 1, according to the manufacturer’s instructions. The thermocycling conditions were as follows: 95°C for 3 min, followed by 30 cycles of 95°C for 20 s, 53°C for 10 s, and 70°C for 45 s, finished with 5 min at 70°C. The products were run on a 2% agarose gel, and required bands were extracted using the QIAquick Gel Extraction Kit (QIAGEN, 28704). To increase the DNA concentration, a second round of PCR was carried out. A 20-μl transcription reaction was set up using 2 μl of the final PCR product, 1× DIG-UTP (digoxigenin-uridine triphosphate) labeling mix (Roche, 000000011277073910), 10 U of T7 or T3 RNA polymerase (Promega, P207B, P208C), 1× polymerase buffer, and 40 U of RNaseOUT RNase inhibitor (Invitrogen, 10777019). The reaction was incubated at 37°C for 2 hours. The sample was treated with 2 U of TURBO DNase (Life Technologies, AM1354) for 20 min at 37°C. Electrophoresis was carried out to confirm whether an intact full-length transcript has been synthesized. The RNA was then precipitated using ammonium acetate. Embryos (24 hpf) were dechorionated and fixed in 4% paraformaldehyde (PFA) in phosphate-buffered saline (PBS) at 4°C overnight and then washed in PBSTw (0.1% Tween 20 in PBS) three times for 5 min. The embryos were then washed through a series of methanol:PBSTw washes of 5 min in each of 25, 50, 75, and 100% methanol in PBSTw and stored at −20°C overnight. The embryos were then rehydrated back into PBSTw in the same series in reverse and then subjected to four 5-min washes in PBSTw. The embryos were treated with proteinase K (10 μg/μl) for 20 min, which was then quenched by two washes with glycine (2 mg/ml) in PBSTw for 5 min. Embryos were fixed in 4% PFA in PBSTw again for 20 min at room temperature and washed five times for 5 min in PBSTw with shaking. Embryos were washed in 50% Hybe^−/−^ [50% formamide, 5× SSC (ChemCruz, SC296419), 9.2 mM citric acid, and 0.1% Tween 20 (pH 6)] in PBSTw for 5 min and then prehybridized in Hybe^+/+^ [50% formamide, 5× SSC, 9.2 mM citric acid, 0.1% Tween 20, tRNA (0.5 mg/ml; Invitrogen, 15401029), and heparin (0.05 g/ml)] at 65°C for at least 1 hour. The pre-Hybe solution was then replaced with 1:200 dilution of the probe in Hybe^+/+^, and the sample was incubated at 65°C overnight. The Hybe solution was aspirated while maintaining the tubes at 65°C. The following 10-min washes at 65°C were carried out: 100% Hybe^−/−^, 75% Hybe in 2× SSCTw (0.1% Tween 20 in 2× SSC), 50% Hybe in 2× SSCTw, 25% Hybe in 2× SSCTw, and 100% 2× SSCTw. The embryos were then washed four times for 15 min in 0.2× SSCTw. The following 5-min washes were executed: 75% 0.2× SSC in MABTw [0.1 M maleic acid, 0.15 M NaCl, and 0.1% Tween 20 (pH7.5)], 50% 0.2× SSC in MABTw, 25% 0.2× SSC in MABTw, and 100% MABTw. The embryos were blocked in 2% Blocking Reagent (Roche, 11096176001) in MABTw for at least 1 hour at room temperature with gentle shaking. The blocking buffer was then replaced with a 1:5000 dilution of α-DIG antibody (Table 2) in blocking buffer, and the sample was incubated overnight at 4°C with gentle rocking. Following this, the sample was rocked for 1 hour at room temperature to complete the antibody reaction. The sample was then washed eight times for 15 min in MABTw with gentle rocking. Embryos were equilibrated in BCL3 developing buffer [100 mM tris (pH 9.5), 100 mM NaCl, 50 mM MgCl_2_, and 0.1% Tween 20] three times for 5 min at room temperature. The developing buffer was then aspirated and replaced with 50% BM Purple (Roche, 11442074001) in BCL3. The staining was developed by gently rocking the embryos at room temperature in tubes wrapped in foil until desired levels of staining were achieved. The reaction was terminated by replacing the BM Purple solution with BCL3 buffer and then fixing in 4% PFA in PBS at least overnight at 4°C. For imaging, samples were washed in a series of 5-min washes: three times in PBSTw, once in 25% glycerol (Invitrogen, 15514-011) in PBSTw, once in 50% glycerol in PBSTw, and once in 70% glycerol in PBSTw. The embryos were imaged on the Leica M165 FC dissecting microscope with the Leica Application Suite version 4.3.0 program.

**Table 1:** List of whole-mount in situ hybridization primers used in this study.

**Target**	**Oligo (uppercase, gene-specific sequence)**	**F/R**	**Tm (°C)**	**Template**	**Product size** **(bp)**
Tdp1	taatacgactcactatagggAGCAGTATCCGCCAGAATTT	F	64.6	cDNA	1030
	aattaaccctcactaaaggTGGTCTCAGCAGCTCAAGAA	R	64.9		

**Table 2:** Antibodies. Species reactivity, host species, supplier, working concentration, and application of primary antibodies. WB, Western blot; SB, slot blot; ISH, in situ hybridization; IgG, immunoglobulin G; HRP, horseradish peroxidase.

**Antibody**	**Species reactivity**	**Host species**	**Supplier (catalog no.)**	**Concentration**	**Application**
DIG	–	Sheep	Roche (11093274910)	1:5000	ISH
Top1-CC	Human, zebrafish	Mouse	Merck (MABE1084)	1:2000	SB
β-Actin	Human, zebrafish	Mouse	Sigma-Aldrich (A5316)	1: 2000	WB
γ-H2AX (Ser^139^)	Zebrafish	Rabbit	GeneTex (GTX127342)	1: 500	WB
IgG (H + L)–HRP conjugate	Mouse	Goat	Bio-Rad (170-6516)	1:4000	WB
IgG (H + L)–HRP conjugate	Rabbit	Goat	Bio-Rad (170-6522)	1:2000 to 1:4000	WB

### Generation of *tdp1^−/−^* zebrafish

CRISPR-Cas9 was performed as described by Hruscha *et al*. (*59*). Single-cell stage embryos were injected with 2.4-μg Cas9 mRNA and single gRNA (sgRNA; 0.4 μg/μl), targeting the first coding exon of zebrafish *tdp1* (Table 3), and raised. Cas9 mRNA was in vitro transcribed from 1 to 2 μg of Not1-linearized pCS2-nCas9n plasmid using the mMESSAGE mMACHINE kit (Life Technologies, AM1340), while sgRNA was transcribed with the MEGAshortscript T7 Transcription Kit (Life Technologies, AM1354), according to the manufacturer’s instructions. Once the injected animals reached sexual maturity, they were outcrossed to wild-type fish, and the progeny were sequenced to identify founders carrying the desired mutations. The selected founders were outcrossed to a wild-type strain to generate *tdp1^−/+^* zebrafish, which were later incrossed to give rise to *tdp1^−/−^* zebrafish. The line was maintained in a mixed London wild-type/nacre background.

**Table 3:** gRNA used in this manuscript.

**Target**	**Sequence**	**Source**
First coding exon of *tdp1* (ENSDARE00001057533) *tdp1*	5′-AAAGCACCGACTCGGTGCCACTTTTTCAAGT TGATAACGGACTAGCCTTATTTTAACTTGCTATT TCTAGCTCTAAAACTTCCTCAGTTTCTCTCTTCCC TATAGTGAGTCGTATTACGC-3′	Integrated DNA Technologies (IDT), Coralville, USA
*apex2*	5′-AGTTTTAGCCGAGGACGAAGTGG-3′	Sigma Merck, Dorset, UK
	5′-ACTCCATTTCTGGCCGAGGAAGG-3′	
	5′-AAGCCATCTTGAGCTCAGGGAGG-3′	
	5′-GAGAGGTGTTCACGTCACCCAGG-3′	

### TDP1 activity assay

TDP1 activity assay was performed similarly to the protocol described by Meisenberg *et al*. (*60*). Embryos (4 dpf) were anesthetized and deyolked in ice-cold PBS by pipetting up and down with a 200-μl pipette tip. The embryos were then washed twice in PBS, homogenized with a micropestle, and lysed in lysis buffer [200 mM Hepes, 40 mM NaCl, 2 mM MgCl_2_, 0.5% Triton X-100, and 1× protease inhibitor cocktail (Roche, 4693159001)] for 30 min on ice. The fin clips were collected, snap-frozen, homogenized, and lysed in lysis buffer. The tissue debris was pelleted at 13,300 rpm for 15 min at 4°C, and the supernatant was collected. Ten to 600 ng of total protein were combined with 1× assay buffer [25 mM Hepes (pH 8.0), 130 mM KCl, and 1 mM dithiothreitol (DTT)] and 2.5 μM (embryos) or 60 nM (fin clips) Cy5.5-labeled substrate oligomer containing a 3′-phosphotyrosyl group (Table 4) in a total volume of 10 μl. The reaction was incubated at 37°C for 1 hour and stopped by the addition of 1× loading buffer (44% deionized formamide, 2.25 mM tris-borate, 0.05 mM EDTA, 0.01% xylene cyanol, and 1% bromophenol blue) and boiling at 90°C for 10 min. The sample was then loaded onto a prerun 20% Urea SequaGel (Fisher Scientific, EC-833-1) and subjected to 150-V electrophoresis for approximately 1 hour. The bands were imaged using the ChemiDoc MP imaging system (Bio-Rad, 1708280).

**Table 4:** TDP1 and TDP2 activity assay oligonucleotides.

**Activity assay**	**Sequence**	**Labels/modifications**	**Source**
TDP1	5′-GATCTAAAAGACT-3′	3′-pY, 5′-Cy5	Midland Certified Reagent Company, TX, USA
TDP2	5′-CATCGTTGCCTACCAT-3′	5′-pY, 3′-Cy5	
	5′-GCATGATGGTAGGCAACGATG-3′	–	IDT

### Locomotion analysis

All zebrafish locomotion analysis was carried out in the Sheffield Zebrafish Screening Unit. During the photomotor response analysis one 5-dpf embryo was added per well of a 24-well plate and acclimatized to the room for 30 min before habituation in 10% light in the ZebraBox Viewpoint system. The larval movement was then recorded during 3 cycles of 5-min darkness (0% light) and 5-min light (10% light) using ZebraLab version 3.20.5.3 software. Adult zebrafish locomotion of up to 10 animals at a time was measured in the ZebraCube (Viewpoint). The data from the first 30 min (TPT/DMSO intraperitoneal injections) or 1 hour (untreated fish movement) were removed to account for acclimatization and were analyzed using ZebraLab software version 3.22.3.9.

Swim tunnel analysis was adapted from the work of Plaut (*61*). Adult zebrafish were habituated in the experiment room for 1 hour before being placed in a transparent water tunnel. The water flow rate in the tunnel was then gradually increased to 6.58 cm/s, maintained for 5 min, and increased in increments of 6.58 cm/s for 5 min at a time until the animals got tired and fell into a mesh at the end of the tunnel. The fish were allowed a second attempt at swimming by reducing the flow rate and then gradually increasing it to the fatigue flow rate for the remainder of the 5-min interval. If the fish got tired again, then the time was recorded when they fell into the mesh. Critical swimming speed, *U*_crit_, was calculated using the formula *U*_crit_ = *U*_i_ + (*U*_ii_*T_i_/*T*_ii_), where *U*_i_ is the highest flow rate sustained for a complete 5-min interval (cm/s), *U*_ii_ is the flow rate increment (6.58 cm/s), *T*_i_ is the time elapsed at the fatigue flow rate (minutes), and *T*_ii_ is the interval of time (5 min).

### Intraperitoneal injections of TPT

Zebrafish were kept in groups of seven to nine and fasted 24 hours before injection. They were anaesthetized with MS-222 (Sigma-Aldrich) and injected intraperitoneally using an insulin syringe with a 30-gauge needle (Bunzl Healthcare, 324826) with TPT hydrochloride (Sigma-Aldrich) or 30% DMSO in sterile Hank’s buffered solution (Gibco, 11530476). A daily dose of TPT hydrochloride (22.5 mg/kg) was injected for two consecutive days for a final concentration of 45 mg/kg in a total volume of 10 μl.

### Measurement of Top1-CCs by fractionation

In vivo complex of enzyme assay was used to purify and quantify Top1-CCs, according to Chiang *et al*. (*62*). Thirty to 40 3-dpf zebrafish embryos or tissues were homogenized and lysed in 1.1-ml lysis buffer [8 M guanidine hydrochloride, 30 mM tris-HCl (pH 7.5), 10 mM EDTA, and 1% sarkosyl (pH 7.5)] for 15 min at 65°C. One-milliliter aliquots of cesium chloride (CsCl) in a range of densities were gently layered on top of each other to form a gradient (from bottom to top: 1.45, 1.5, 1.72, and 1.82 g/ml) in a 5-ml polyallomer centrifuge tube (Beckman, 326819). The lysate was centrifuged at 16,000*g* for 10 min; then, 1 ml of the supernatant was carefully layered on top of the CsCl gradient. The layered sample was centrifuged at 30,000 rpm in a Beckman Ultima LE-80K ultracentrifuge with a swinging rotor for 24 hours at 25°C and stopped gradually without a brake. In the meantime, 10 μl of the remaining lysate was made up to 100 μl in 1× TE buffer [10 mM tris and 1 mM EDTA (pH 8)] and incubated with RNase A (ribonuclease A; 0.5 μg/ml) at 37°C overnight. Fifty microliters of the RNase-digested sample or 1× TE buffer was mixed with an equal volume of 1× TE buffer with a 1:200 dilution of PicoGreen (Invitrogen, P7581). λ DNA standard (5 μg/μl) was also mixed with 1× TE buffer with PicoGreen, and a serial dilution was carried out to obtain a range of DNA standards (125 ng to 5 μg). DNA concentration was quantified in a FLUOstar Omega microplate reader (BMG) with fluorescence at EX485-12/EM520. Fractionated lysates were collected by piercing the bottom of the tube with a 19-gauge syringe needle (positioned at 45° with the bevel upward), connected to a Pharmacia Biotech P-1 peristaltic pump with a silicone tube. Each sample was collected in 10 fractions of 0.5 ml. Fractions with equal double-stranded DNA amounts between samples (maximum, 200 μl) were subjected to slot blotting onto a PBS-wetted 0.45-μm nitrocellulose membrane (GE Healthcare, 106000002). The membrane was air-dried and subjected to immunoblotting with a 1:2000 dilution of a Top1-CC antibody (Table 2).

### Western blotting

Zebrafish embryos were deyolked by trituration in PBS, then homogenized, and lysed in 1 to 1.5 μl of lysis buffer [200 mM Hepes, 40 mM NaCl, 2 mM MgCl_2_, 0.5% Triton X-100, and 1× protease inhibitor cocktail (Roche)] per embryo. Total protein concentration was determined using Bradford reagent (Bio-Rad); then, 100 μg per lane were run on a 15% SDS–polyacrylamide gel electrophoresis gel and transferred onto a 0.45-μm nitrocellulose membrane (Bio-Rad, 170-4271) using the Trans-Blot Turbo Transfer System (Bio-Rad, 17001915), according to the manufacturer’s instructions. The nitrocellulose membrane was blocked in blocking buffer [5% milk, 200 mM tris, 140 mM NaCl, and 0.1% Tween 20 (pH 7.4)] for 1 hour at room temperature and then incubated at 4°C overnight with the primary antibody in blocking buffer. The membrane was then washed three times for 5 min in 1× TBST buffer [200 mM tris, 140 mM NaCl, and 0.1% Tween 20 (pH 7.4)] and incubated for 1 hour with horseradish peroxidase–conjugated secondary antibody in blocking buffer at room temperature. The three washes were repeated before adding the Clarity Western ECL blotting substrate (Bio-Rad, 1705060) onto the membrane. Bands were visualized in the ChemiDoc MP imaging system (Bio-Rad, 1708280) and quantified using Image Lab version 4.1 (Bio-Rad) software. Details of antibodies are provided in Table 2.

### TDP2 activity assay

Cy5.5-labeled substrate oligomer (100 pmol) was combined with 100 pmol of a 20-bp complementary oligonucleotide with a 5′ overhang in a total volume of 33.3 μl (Table 4). The sample was denatured at 95°C for 5 min and reannealed by dropping the temperature by 2°C/s for 5 s and then by 0.1°C/s for 600 s. Once annealed, 3 μM double-stranded substrate oligomer with a 5′ overhang was generated. We hypothesized that such a substrate should prevent ligation of product by zebrafish RNA ligases and, thus, undesirable full repair. Zebrafish were deyolked and lysed as described for the TDP1 activity assay. A total of 0.5, 1, and 5 μg of the lysate were combined with 1× TDP2 activity assay buffer [5 mM tris (pH 7.5), 5 mM KCl, 0.1 mM DTT, bovine serum albumin (10 μg/ml), and 0.1 mM MgCl_2_], 60 nM Cy5.5-labeled substrate oligomer, and 2 μM competitor oligo. The bands were imaged using the ChemiDoc MP imaging system (Bio-Rad, 1708280).

### Reverse transcription qPCR

Thirty-five to 45 zebrafish embryos were homogenized in TRIzol reagent (Invitrogen), and total RNA was extracted, according to the manufacturer’s instructions. The NanoDrop (Thermo Fisher Scientific) was used to quantify RNA; then, 1 μg of RNA was reverse-transcribed using the High-Capacity cDNA Reverse Transcription Kit (Applied Biosystems). cDNA from all conditions was pooled, serially diluted to 20, 4, 0.8, and 0.16%, and run alongside unknown samples to serve as a standard curve for extrapolating the sample concentration. Two microliters of a 1:16 dilution of individual cDNA was used in a 20-μl qPCR reaction with SensiMix SYBR no-ROX master mix (Bioline) in a Rotor-Gene 6000 real-time thermocycler (Corbett Research) under the following conditions: 95°C for 10 min, followed by 40 to 45 cycles of 95°C for 15 s, 55°C for 15 s, and 72°C for 15 s (Table 5). All samples were run in duplicate and normalized to *rps29*. Quantification was performed using the Rotor-Gene Q version 2.3.5 software. Primer sequences and other details are listed in Table 5.

**Table 5:** RT-qPCR probes.

**Name**	**Sequence (5′-3′)**	**F/R**	**Product size (bp)**	**Target** **exon(s)**	**Targeted splice variants**	**No. of** **qPCR** **cycles**	**Source**
*rps29*	TTTGCTCAAACCGTCACGGA	F	110	2, 3	ENSDART00000060444.6 (1/1)	Same as target gene	Bower *et al*. (*66*)
	ACTCGTTTAATCCAGCTTGACG	R					
*tdp2b*	TTGAAGACGGACAATGCGGA	F	128	2, 3	ENSDART00000103612.5 (1/1)	45	This manuscript
	AGCTTGCTGTCCTCCACTTC	R					
*mre11a*	CTTCAGTGTGCATGGCAACC	F	104	5, 6	ENSDART00000163434.3 ENSDART00000157758.2 (2/2)	40	This manuscript
	GACTGCGGCCGAAATGATTC	R					
*mus81*	CAGAAAGGCCTGCAGTAGCT	F	102	8	ENSDART00000100782.6 ENSDART00000190027.1 (2/5)	45	This manuscript
	CTGTCCTCCCGGTTTCTGAC	R					
*ercc4*	ACCAATCCCAGGAGAGAACG	F	93	7, 8	ENSDART00000193248.1 ENSDART00000015780.8 (2/2)	45	This manuscript
	AGAATGGTTTAGCGGGGTCA	R					
*ercc1*	CGATTCAGCGTTCTCAAAGGA	F	151	1, 2	ENSDART00000041751.7 (1/1)	40	N. Li
	CTGATGGCCCTTGTGTTTGT	R					
*apex2*	TGAACACCTCTCACAGACCC	F	86	4, 5 (202) 5, 6 (201)	ENSDART00000189272.1 ENSDART00000021514.7 (2/2)	40	This manuscript
	ATCCAGCCACTTTCTCCCAG	R					
*apex2*_201	TCCTGGATTCATTTGATGCGG	F	62	5, 6	ENSDART00000021514.7 (1/2)	40	This manuscript
	CAGGTCGCGGGTAACTTTG	R					
*rbbp8*	AGGAGCTGATCTCGGTCAGT	F	80	12, 13	ENSDART00000063832.6 (1/2)	45	This manuscript
	CCAGCGTCTCCAAGTCTGTT	R					
*sprtn*	CGATGAACAGACCTCCCTCG	F	150	4	ENSDART00000158057.2 (1/1)	40	This manuscript
	TCTCAGTGGCAGGCATCTTG	R					
*neil1*	TTGATGAACGGCAGTCCCAG	F	170	2	ENSDART00000014052.9 (1/2)	40	This manuscript
	GCCAAATCTGCGGGTATCCT	R					
*tdp1*	GCTCCTCAATTGGCTTCCCT	F	133	12, 13	ENSDART00000150149.3 (1/1)	45	This manuscript
	ATGTTCCAGATCCAA GGCCG	R					

### Microarray

Zebrafish embryos were homogenized in TRIzol reagent (Invitrogen) to extract RNA, according to the manufacturer’s protocol. RNA was then sent to the Affymetrix Microarray Core Facility at the University of Sheffield. There, samples were processed using the Affymetrix WT plus protocol. Total RNA (200 ng) was taken forward to biotin labeling. Samples were hybridized using the Affymetrix standard protocol and the hybridization, wash, and stain solutions. Hybridization was carried out overnight at 45°C for 16 hours in a rotating hybridization platform. Post-hybridization stringency washing was performed using the Affymetrix fluidics station, following the protocol outlined in the Affymetrix instructions. The samples were scanned on an Affymetrix GeneChip Scanner 3000, according to the manufacturer’s protocols. Raw data were processed and Robust Multi-array Average (RMA) normalized using the Transcriptome Analysis Console version 4.0 (Applied Biosystems).

### *Apex2* crispants

Wild-type embryos at one-cell stage and under were injected with a pool of four gRNAs against genomic *apex2* (Table 3). Each embryo was injected with 1 nl of a solution containing of 2.5 fmol of each gRNA (total, 10 fmol), 10 fmol of Cas9 protein (NEB, M0386), and 10 fmol of trans-activating CRISPR RNA (tracrRNA). gRNA efficiency was determined by PCR on DNA extracted from single embryos. DNA extraction was carried out using the HotSHOT DNA isolation method. A total of 25 μl of 1× base solution (1.25 M KOH crystals and 10 mM EDTA) was added to each embryo and incubated for 30 min at 95°C. Tubes were vortexed, and 25 μl of 1× neutralization solution (2 M tris-HCl in Milli-Q water) was added before vortexing again; the DNA was kept on ice. The solution was centrifuged for 2 min at 4200 rpm. PCR was carried out using primers listed in Table 6. Embryo images were taken using the Leica M165 FC dissecting microscope with Leica Application Suite version 4.3.0; total distance traveled was quantified using ImageJ, as described by Meijering *et al*. (*63*), after correcting for camera movement.

**Table 6:** *apex2* genotyping primers.

**Primer**	**Sequence (5′-3′)**	**F/R**	**Source**
*Apex2*	ACAACCTCATGTTGCCCATAAC	F	IDT
	TGGTCACCATAGCAACCAATAA	R	

### Statistical analyses

For adult behavioral analysis, *n* = 36 corresponds to each of the 18 fish recorded twice. *P* values were calculated using two-tailed Student’s *t* test with Holm adjustment for multiple comparisons. For embryonic behavioral analysis, *P* values were calculated between *tdp1^−/−^* and *tdp1^−/+^* pairs from *n* = 39 (4 dpf) or *n* = 14 (5 dpf) for *tdp1^−/+^* DMSO, *n* = 49 (4 dpf) or *n* = 25 (5 dpf) for *tdp1^−/−^* DMSO, *n* = 43 (4 dpf) or *n* = 15 (5 dpf) for *tdp1^−/+^* CPT, and *n* = 45 (4 dpf) or *n* = 22 (5 dpf) for *tdp1^−/−^* CPT using a two-tailed Student’s *t* test with Holm adjustment for multiple comparisons. In the swim tunnel test, *n* = 6 and the *P* value for the “survival” curve was calculated using the Mantel-Cox test, while for the *U*_crit_, weight, and length parameters, a two-tailed Student’s *t* test was used; for the drop test (*n* = 8), *P* values were calculated by two-tailed Student’s *t* test. For intraperitoneal TPT injections, *n* = 7 for TPT-treated and DMSO-treated *tdp1^WT^* and DMSO-treated *tdp1^−/−^*, while *n* = 8 for TPT-treated *tdp1^−/−^*. *P* values were calculated using two-tailed Student’s *t* test with Holm post hoc analysis for multiple comparisons; for the blind CPT sensitivity assay in embryos, *n* = 40 for *tdp1^−/+^* incross and *n* = 23 for the *tdp1^−/−^* and *tdp1^−/+^* cross. A chi-square test was used to calculate two-tailed *P* value; for Top1-CC quantification and Western blots, *P* values were calculated from three biologically independent experiments with two-tailed Student’s *t* test. For γH2AX, *n* = 8 for all untreated conditions, *n* = 10 for IR0 *tdp1^WT^* cerebellum, *n* = 8 for IR0 *tdp1^−/−^* cerebellum, *n* = 8 for IR0 *tdp1^WT^* optic tectum, *n* = 9 for IR0 *tdp1^−/−^*optic tectum, *n* = 5 for IR30 *tdp1^WT^* cerebellum and optic tectum, and *n* = 4 for IR30 *tdp1^−/−^* cerebellum and optic tectum. qPCR results were quantified from three biological repeats and normalized to *rps29*. *P* values were derived from performing two-way analysis of variance (ANOVA) with Holm post hoc analysis for multiple comparisons. The microarray experiment was performed on samples from three biologically independent experiments, and results were processed using the Transcriptome Analysis Console version 4.0 with default analysis settings, except for fold change (<−1.2 or >1.2). The *apex2* crispant movement analysis was performed on *n* = 33 for control uninjected DMSO-treated embryos, *n* = 47 for control uninjected *apex2* crispants, *n* = 35 for control uninjected CPT-treated embryos, and *n* = 39 for CPT-treated *apex2* crispants; *P* values were calculated using two-way ANOVA with Sidak post hoc analysis. All values were plotted ±SEM. *P* values are indicated as follows: not significant, *P* > 0.05; *P < 0.05; **P < 0.01; ***P < 0.001; and ****P < 0.0001.

## SUPPLEMENTARY MATERIALS

Supplementary material for this article is available at http://advances.sciencemag.org/cgi/content/full/7/5/eabc4165/DC1

## Appendix

View/request a protocol for this paper from *Bio-protocol*.
